# Progress in Surgery

**Published:** 1898-10-15

**Authors:** 


### Progress in Surgery.

### SURGERY OF THE ŒSOPHAGUS AND STOMACH.

**Œsophageal Pouch.**—A case of this condition is recorded by Wright and Smith,[1] in a man forty-five years of age, who had had difficulty in swallowing for ten years, and since that time had noticed that after he has finished eating some food lodges in his throat and is regurgitated. After a meal overnight he could always bring up some of the food next morning, and by pressure on the right side of the neck, just above the sterno-clavicular joint, he could force the food up into the mouth, and such pressure caused air to escape with a squeaking noise. His mother was similarly affected. A truss was tried to press upon and obliterate the sac, but he was unable to wear it. By systematically emptying the pouch each time after eating and drinking the patient got along fairly comfortably without operation.

**Total Extirpation of the Stomach.**—Dr. Brigham[2] has published a second case, which was as radical in the operative procedure employed as the well-known case of Schlatter.[3] Brigham's patient was a woman, aged sixty-six years. After the stomach had been removed, he found that the ends of the œsophagus and duodenum could be brought together without undue tension. As the pulse was weak, they were united by the Murphy button (œsophago-duodenostomy). In Schlatter's case this was not possible, and he therefore united the œsophagus laterally to the intestine about twelve inches below the duodenojejunal fold (œsophago-jejunostomy). In Brigham's case the growth was at the pyloric end, and involved more than half the stomach. The patient made a good recovery. Water was given by the mouth the day after operation, and other fluids were then administered. Solid food was given on the eighteenth day, and seven weeks after the operation the patient was gaining weight, and was able to walk. At no time was undigested food passed in the motion, which agrees with Schlatter's case. Schuchardt[4] says in reference to this subject of gastrectomy that stomach carcinoma appears in three different forms; the first spreads quickly, attaching itself early to the surrounding tissues, and making a radical operation very difficult, while the other two, the infiltrating and tumour forms, can often be easily removed after having existed a long time. Hoffman,[5] who is attached to Eichhorst's clinic, has investigated the metabolism in Schlatter's case of total resection of the stomach. He found at first that there was a retention of nitrogen, but afterwards with a more varied diet this disappeared, showing that the regeneration of the blood was so far complete that there was no longer any need of retention of nitrogen. He has also investigated the action of hydrochloric acid upon putrefactive processes, and concludes that the absence of the acid in this case was without influence upon the putrefactive processes in the alimentary canal. He adds that the administration of hydrochloric acid as a disinfectant of the intestinal contents is useless. The quantity of chlorides in the urine after a meal was not decreased, which confirms the current view that the diminution of the chlorides in the urine after a meal is due to the hydrochloric acid excreted by the stomach. The urine was examined for the presence of pepsin, but no trace was found.

1 Brit. Med. Jour., April 9, 1898. 2 Lancet, July 2, 1898; and Boston Med. and Surg. Jour., May 5, 1898. 3 Lancet, Jan. 15, 1898. 4 New York Med. Rec., May 7, 1898, p. 677. 5 Münch. Med. Woch, May 3. 1898; and Brit. Med. Jour., June 11, 1898.

